# Supplementary material for: Quantitative Ethnobotany of Medicinal Plants from Darjeeling District of West Bengal, India, along with Phytochemistry and Toxicity Study of Betula alnoides Buch.-Ham. ex D.Don bark
Source: Plants (Basel). 2024 Dec 16;13(24):3505. doi: 10.3390/plants13243505 (PMC11679848; doi:10.3390/plants13243505)
Supplement: Supplementary file 1 [file plants-13-03505-s001.zip › plants-3142538-supplementary.pdf]

Supplementary Table 1: A list of the informants interviewed, along with the GPS coordinates of their residences and plant collection areas.

Sl. No.	Name	Age (yrs)	Sex	Ethnicity	Category of informant	Village, Block	Latitude and Longitude of residential area	Latitude and Longitude of plant collection area
1.	Abishek Mangar	37	Male	Thapa	KP	Lanku, Kurseong	N 26°56'58'' E 88°25'05''	N 26°56'52'' E 88°25'15''
2.	Balaram Khawas	78	Male	Khawas	KP	Shelpu, Kurseong	N 26°56'10'' E 88°23'55''	N 26°56'12'' E 88°23'53''
3.	Binod Chamling	52	Male	Rai	KI	Upper Rolok, Kurseong	N 26°56'50'' E 88°25'07''	N 26°56'48'' E 88°10'05''
4.	Basant Thapa	70	Male	Thapa	KP	Lanku, Kurseong	N 26°55'52'' E 88°24'01''	N 26°55'53'' E 88°24'11''
5.	C.B. Thapa	45	Male	Thapa	KP	Lanku, Kurseong	N 26°56'58'' E 88°25'05''	N 26°57'53'' E 88°25'17''
6.	Devmaya Chamling	66	Female	Rai	KP	Turzum, Jorebunglow Sukiapokhri	N 26°56'3'' E 88°10'40''	N 26°56'3'' E 88°10'11''
7.	Dhurba Mangar	60	Male	Thapa	KI	Lanku, Kurseong	N 26°57'01'' E 88°25'07''	N 26°57'50'' E 88°25'15''
8.	Dipal Rai	51	Male	Rai	KI	Mungpoo, Kurseong	N 26°58'55'' E 88°23'34''	N 26°58'54'' E 88°23'31''
9.	Gauri Chettri	55	Male	Chettri	KI	Samripan, Jorebunglow Sukiapokhri	N 26°57'20'' E 88°11'55''	N 26°57'21'' E 88°11'54''
10.	Hemraj Sharma	67	Male	Sharma	KI	Rageroon, Jorebunglow Sukiapokhri	N 27°01'02'' E 88°17'06''	N 27°01'01'' E 88°17'04''

11.	Hombahadur Mangar	45	Male	Thapa	KP	Lanku, Kurseong	N 26°56’58’’ E 88°25’05’’	N 26°56’53’’ E 88°25’11’’
12.	Jagat Mangar	55	Male	Thapa	KI	Lanku, Kurseong	N 26°57’03’’ E 88°25’06’’	N 26°58’04’’ E 88°25’11’’
13.	J.B. Limbu	73	Male	Limbu	KP	Upper Sawreni, Mirik	N 26°52’34’’ E 88°11’26’’	N 26°52’35’’ E 88°11’28’’
14.	Jhagare Rai	66	Male	Rai	KP	Lanku, Kurseong	N 26°56’58’’ E 88°25’05’’	N 26°56’52’’ E 88°25’11’’
15.	Jiten Subba	56	Male	Limbu	KP	Samripan, Jorebunglow Sukiapokhri	N 26°57’24’’ E 88°11’51’’	N 26°57’22’’ E 88°11’52’’
16.	Jiwan Thapa	67	Male	Thapa	KI	Tarzum, Jorebunglow Sukiapokhri	N 26°56’3’’ E 88°10’40’’	N 26°56’3’’ E 88°10’11’’
17.	Kalyan Rai	55	Male	Rai	KP	Sittong, Jorebunglow Sukiapokhri	N 26°55’43’’ E 88°23’54’’	N 26°55’41’’ E 88°23’54’’
18.	Kalpana Khawas	53	Female	Khawas	KP	Sittong, Kurseong	N 26°55’43’’ E 88°23’54’’	N 26°55’41’’ E 88°23’53’’
19.	Leela Subba	60	Female	Limbu	KP	Batar, Kurseong	N 26°56’24’’ E 88°24’11’’	N 26°55’41’’ E 88°23’54’’
20	Manbahadur Limbu	58	Male	Limbu	KP	Rangli, Rangli Rangliot	N 26°57’24’’ E 88°11’52’’	N 26°57’22’’ E 88°11’51’’
21.	Marpo Tshering Lepcha	34	Male	Lepcha	KI	Lanku, Kurseong	N 26°56’50’’ E 88°25’01’’	N 26°56’52’’ E 88°25’15’’
22.	Manjeet Subba	60	Male	Limbu	KP	Rangli, Rangli Rangliot	N 26°57’24’’ E 88°11’52’’	N 26°57’22’’ E 88°11’51’’

23.	Mankala Limbu	67	Female	Limbu	KP	Upper Sawreni, Mirik	N 26°52’34’’ E 88°11’26’’	N 26°52’35’’ E 88°11’28’’
24.	Meena Mangar	58	Female	Thapa	KP	Lanku, Kurseong	N 26°56’58’’ E 88°25’05’’	N 26°56’52’’ E 88°25’15’’
25.	Nawraj Sharma	50	Male	Sharma	KI	Phuguri, Mirik	N 26°50’44’’ E 88°12’30’’	N 27°51’47’’ E 89°13’31’’
26.	Nagen Khawas	45	Male	Khawas	KP	Shelpu, Kurseong	N 26°56’11’’ E 88°23’54’’	N 26°56’13’’ E 88°23’52’’
27.	Pasang Lepcha	51	Male	Lepcha	KI	Lanku, Kurseong	N 26°56’54’’ E 88°25’01’’	N 26°57’50’’ E 88°25’15’’
28.	Pema Lahmu Sherpa	40	Female	Sherpa	KP	Near 6th Mile, Rangli Rangliot	N 27°01’20’’ E 88°18’37’’	N 27°02’21’’ E 88°18’38’’
29.	Prem Khawas	69	Male	Khawas	KP	Namthing, Kurseong	N 26°55’43’’ E 88°23’54’’	N 26°55’41’’ E 88°23’54’’
30.	Prakash Subba	40	Male	Limbu	KP	Batar, Kurseong	N 26°55’52’’ E 88°23’50’’	N 26°55’39’’ E 88°23’59’’
31.	Sabita Rai	69	Female	Rai	KP	Rolok, Kurseong	N 26°57’11’’ E 88°24’43’’	N 26°57’15’’ E 88°24’45’’
32.	Rabin Tamang	55	Male	Tamang	KP	Shelpu, Kurseong	N 26°56’11’’ E 88°23’54’’	N 26°56’12’’ E 88°23’52’’
33.	Raksha Limbu	35	Female	Limbu	KP	Lanku, Kurseong	N 26°57’24’’ E 88°11’52’’	N 26°57’22’’ E 88°11’51’’
34.	Rakchak Khawas	32	Female	Khawas	KP	Shelpu, Kurseong	N 26°56’11’’ E 88°23’55’’	N 26°56’13’’ E 88°23’57’’
35.	Ranjit Subba	31	Male	Limbu	KP	Sittong, Kurseong	N 26°56’11’’	N 26°56’11’’

							E<br>88°24’24’’	E<br>88°24’54’’
36.	Sahadev Rai	72	Male	Rai	KP	Rolok,<br>Kurseong	N<br>26°57’11’’<br>E<br>88°24’43’’	N<br>26°57’15’’<br>E<br>88°24’45’’
37.	Sanju Lepcha	39	Female	Rai	KP	Lanku,<br>Kurseong	N<br>26°56’50’’<br>E<br>88°25’01’’	N<br>26°56’48’’<br>E<br>88°25’02’’
38	Sankar Chettri	42	Male	Chettri	KP	Rangli,<br>Rangli<br>Rangliot	N<br>26°55’10’’<br>E<br>88°23’52’’	N<br>26°57’10’’<br>E<br>88°21’55’’
39.	Sardha Rai	34	Female	Rai	KP	Lanku,<br>Kurseong	N<br>26°56’50’’<br>E<br>88°25’01’’	N<br>26°56’52’’<br>E<br>88°25’03’’
40.	Sarala Subba	51	Female	Limbu	KP	Samripani,<br>Jorebunglow<br>Sukiapokhri	N<br>26°57’24’’<br>E<br>88°11’51’’	N<br>26°57’22’’<br>E<br>88°11’52’’
41.	Shewan Rai	42	Male	Rai	KP	Sittong,<br>Kurseong	N<br>26°56’11’’<br>E<br>88°24’24’’	N<br>26°56’11’’<br>E<br>88°24’54’’
42.	Sudeep Tamang	35	Male	Tamang	KI	Mungpoo,<br>Kurseong	N<br>26°58’14’’<br>E<br>88°22’12’’	N<br>26°58’13’’<br>E<br>88°22’11’’
43.	Sushma Rai	34	Female	Rai	KP	Rageroon,<br>Jorebunglow<br>Sukiapokhri	N<br>27°01’02’’<br>E<br>88°17’06’’	N<br>27°01’01’’<br>E<br>88°17’04’’
44.	Saroj Rai	48	Male	Rai	KP	Lanku,<br>Kurseong	N<br>26°56’50’’<br>E<br>88°25’01’’	N<br>26°56’51’’<br>E<br>88°25’03’’
45	Samjana Rai	35	Female	Rai	KP	Rageroon,<br>Jorebunglow<br>Sukiapokhri	N<br>27°01’01’’<br>E<br>88°17’05’’	N<br>27°01’01’’<br>E<br>88°17’04’’
46.	Sushmita Chettri	31	Female	Chettri	KP	Rageroon,<br>Jorebunglow<br>Sukiapokhri	N<br>27°01’02’’<br>E<br>88°17’06’’	N<br>27°01’01’’<br>E<br>88°17’04’’
47.	Swarnima Rai	30	Female	Rai	KP	Samripani,<br>Jorebunglow<br>Sukiapokhri	N<br>26°57’24’’<br>E<br>88°11’51’’	N<br>26°57’22’’<br>E<br>88°11’52’’

[KP= Knowledgeable persons; KI= Key Informant]

Supplementary Table 2: Mortality percentage and CC_{50} value of methanolic bark extract of *B. alnoides* against the L929 mouse fibroblast cell line.

**Concentration (mg/L)**	**Mortality (%)**			**Mean value**	**Standard Deviation**
	**Experiment 1**	**Experiment 2**	**Experiment 3**		
50	10.8	11.1	10.21	10.8	0.4528
100	21.2	20.25	21.08	21.08	0.5173
150	31.03	30.21	31.1	31.03	0.4948
200	40.8	39.47	40.21	40.21	0.6664
250	45.59	44.12	45.11	45.11	0.7496
300	54.11	55.14	54.17	54.17	0.5781
**CC_{50} value**	269.02	271.65	270.07	270.07	1.3238
